# Premature senescence of cardiac fibroblasts and atrial fibrosis in patients with atrial fibrillation

**DOI:** 10.18632/oncotarget.19853

**Published:** 2017-08-03

**Authors:** Jun Xie, Yuhan Chen, Chuanxian Hu, Quanhua Pan, Bingjian Wang, Xueling Li, Jin Geng, Biao Xu

**Affiliations:** ^1^ Department of Cardiology, Drum Tower Hospital, Nanjing University Medical School, Nanjing, Jiangsu, China; ^2^ Department of Thoracic and Cardiovascular Surgery, Huai’an First People's Hospital, Nanjing Medical University, Huai’an, Jiangsu, China; ^3^ Department of Cardiology, Huai’an First People's Hospital, Nanjing Medical University, Huai’an, Jiangsu, China

**Keywords:** premature senescence, P16, cardiac fibroblast, fibrosis, atrial fibrillation, Gerotarget

## Abstract

Premature senescence is associated with atrial fibrosis and has an antifibrotic effect in mice. However, the role of senescence in atrial fibrillation (AF) remains unclear. Here, we investigated the association of premature senescence with fibrosis and also determined the role of senescence in the recurrence of AF after surgery ablation. Western blot, Sirius red staining, SA-β-gal staining and immunohistochemistry were performed to detect the degree of atrial fibrosis ,the expression of TGF-β and collagens, and also the senescence markers in 72 tissue specimens of left atrial appendage in this study. Then the patients undergoing successful surgical ablation were followed up for 12 months. The expression of collagens and TGF-β was paralleled by a high level of atrial fibrosis and were increased in AF group, especially in the persistent AF group. Western blotting of P16 and SA-β-gal staining showed an increased premature senescence in the sinus rhythm, paroxysmal AF and persistent AF groups. In addition, positive area of senescence markers, SA-β-gal and P16, was correlated positively with fibrotic lesions. We also found a lower ratio of P16/TGF-β in patients with recurrence of AF than in patients without recurrent AF. In conclusion, premature senescence is associated with atrial fibrosis in AF, and may have an antifibrotic role in AF.

## INTRODUCTION

Atrial fibrillation (AF) has become a serious epidemic across the world, and the incidence is expected to double within the next 20 years [1–3]. Although there is considerable progression in the diagnosis and treatment of AF, it is associated with increased morbidity and mortality [2]. It is generally known that atrial fibrosis contributes to atrial structural remodeling, leading to the development and maintenance of AF [4, 5]. However, the underlying mechanisms of fibrosis in AF remain unclear.

The prevalence of AF is age dependent [2, 3]. Moreover, short telomere length is a hallmark of aging and is associated with the incidence of AF [6], indicating it as a major risk factor for AFs. Replicative senescence appears to be a fundamental role in aging, which is characterized by DNA damage and telomere erosion, contributing to cardiomyocyte hypertrophy, increased apoptosis, decreased myocyte number and myocardial fibrosis [7, 8]. Whereas premature senescence is an irreversible form of cell-cycle arrest and primarily designed to initiate the elimination of damaged cells [7]. In addition, P16 and P21 were up-regulated in senescent cells and senescence-associated β-galactosidase (SA-β-gal) distinguishes them from quiescent cells [9].

According to a recent study by Meyer senescence of cardiac fibroblasts (CFs) plays an essential antifibrotic role in murine fibrotic model [10]. Similar results were obtained in the heart biopsies of patients with idiopathic cardiomyopathy, and showed a positive relationship between senescence and fibrosis [10]. These findings suggest that senescence may be associated with atrial fibrosis in the development of AF. We therefore, analyzed human atrial biopsies from AF patients to characterize the association of premature senescence and fibrosis and also to determine the role of senescence in the recurrence of AF after surgery ablation.

## RESULTS

### Patient characteristics

Table 1 presents the baseline characteristics of included individuals. Left atrial diameter (LAD) was significantly increased in the AF groups compared to sinus rhythm (SR) group, and it was larger in persistent AF (PeAF) group than in paroxysmal AF (PaAF) group. Besides, pulmonary artery systolic pressure (PASP) was observed to be highest in the PeAF group, followed by PaAF and SR groups. However, no significant difference was observed between PeAF and PaAF groups. No statistical significance was observed among the groups for other echocardiographic parameters, basic data and preoperation drugs.

**Table 1:** Baseline characteristics of included patients

Parameters	SR (*n*= 26	PaAF (*n*= 17)	PeAF (*n*= 29)	*P* value
Basic data				
Gender (M/F), *n*	13/13	7/10	11/18	0.655
Age, y	57.3±10.4	58.9±7.3	59.9±8.5	0.548
BMI, kg/m^2^	24.6±2.9	24.3±2.6	25.0±3.3	0.732
NYHA class	2.6±0.6	2.6±0.7	2.6±0.7	0.891
ECG parameters				
LVEDd, cm	5.0±0.8	5.1±0.5	5.2±0.9	0.559
LVESd, cm	4.1±0.8	4.3±0.5	4.4±0.8	0.355
LAD, cm	3.6±0.7	4.8±0.9 ***	5.4±1.0 ***^, #^	<0.001
LVEF, %	59.6±11.9	56.9±7.0	55.2±7.9	0.220
PASP, mmHg	36.2±7.0	45.7±7.8 **	49.4±7.0 **	0.002
Preoperative drugs				
ACEI/ARB, *n*	7	4	4	0.465
β-blockers, *n*	9	4	6	0.481
CCB, *n*	3	3	4	0.852
Digoxin, *n*	15	11	24	0.117
Spirolactone, *n*	5	5	13	0.123

SR, sinus rhythm; PaAF, paroxysmal atrial fibrillation; PeAF, persistent atrial fibrillation; BMI, body mass index; NYHA, New York Heart Association; ECG, echocardiograph; LVEDd, left ventricular end-diastolic diameter; LVESd, left ventricular end-systolic diameter; LAD, left atrial diameter; EF, left ventricular ejection fraction; PASP, pulmonary artery systolic pressure; ACEI, angiotensin converting enzyme inhibitor; ARB, angiotension receptor blocker; CCB, calcium channel blocker. **P < 0.01 *vs* SR group. ***P < 0.001 *vs* SR group. #, <0.01 *vs* PaAF group.

### Up-regulated fibrosis in AF

**Figure 1:**
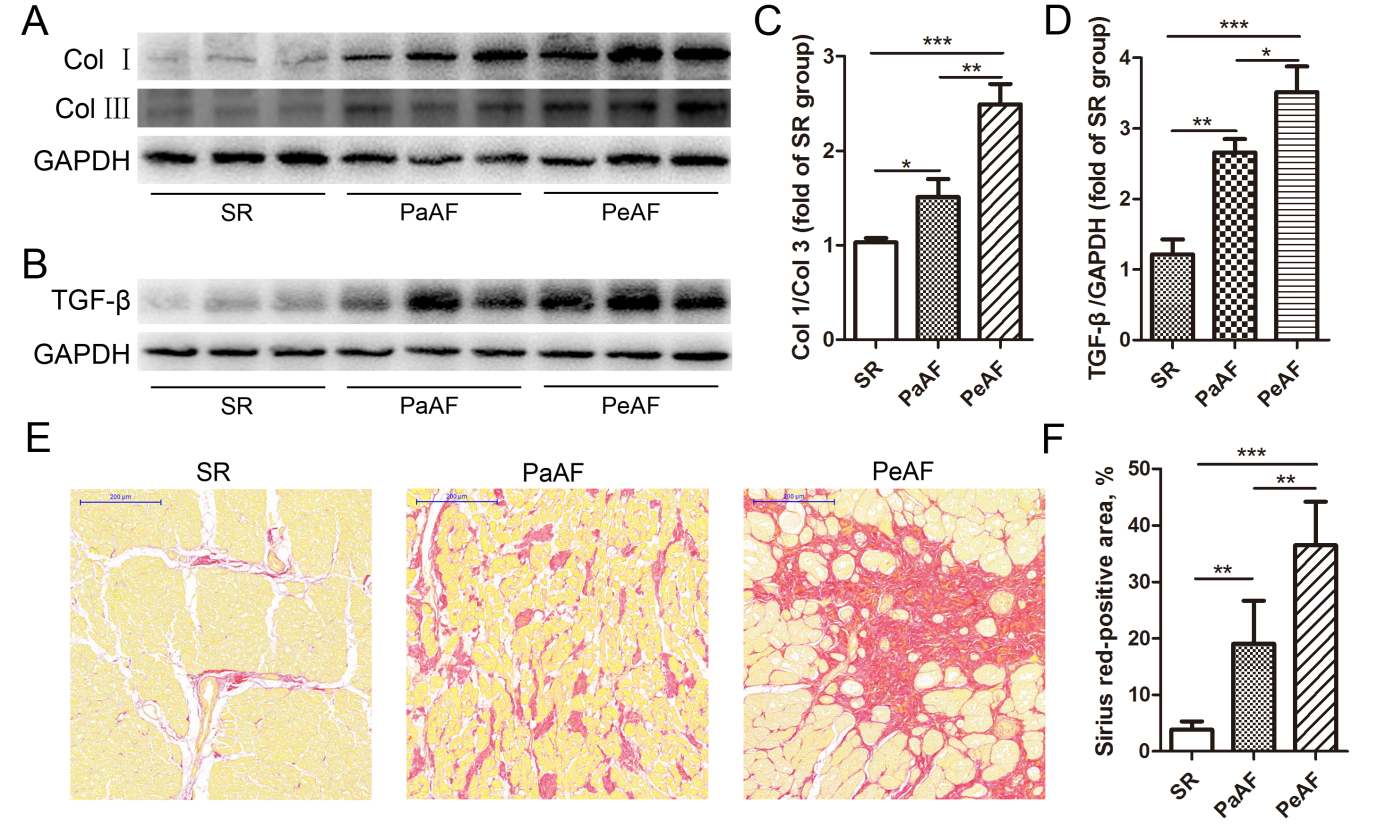
Up-regulation of atrial fibrosis in AF **A**. & **B**., The expression of Col I, Col III and TGF-β were increased in the AF groups. **C**.&**D**., Statistical analyses of the ratio of Col I/Col III and TGF-β (*n* = 3 per group). **E**., Sirius red staining showed a gradient increase of atrial fibrosis in SR, PaAF and PeAF groups; bar = 200μm. **F**., Quantification of Sirius red positive area in the three groups (*n* = 5 per group). Values were expressed as mean ± SD. *P < 0.05, **P < 0.01, ***P < 0.001.

Western blotting analysis suggested that Col I and Col III were gradually and significantly increased in the SR, PaAF and PeAF groups. The highest ratio of Col I/ Col III was observed in the PeAF group, while the lowest ratio in the SR group (Figure 1A and 1C). Similarly, the expression of TGF-β was highest in the PeAF group, followed by the PaAF and SR groups (Figure 1B and 1D). Then, as depicted in Figure 1E and 1F, the positive Sirius red stained area was significantly higher in the AF group than in the SR group. In the two AF groups, the percentage of interstitial fibrosis was higher in the PeAF group compared to PaAF group (Figure 1E and 1F).

### Accumulation of senescence accompanied by fibrosis in AF

**Figure 2:**
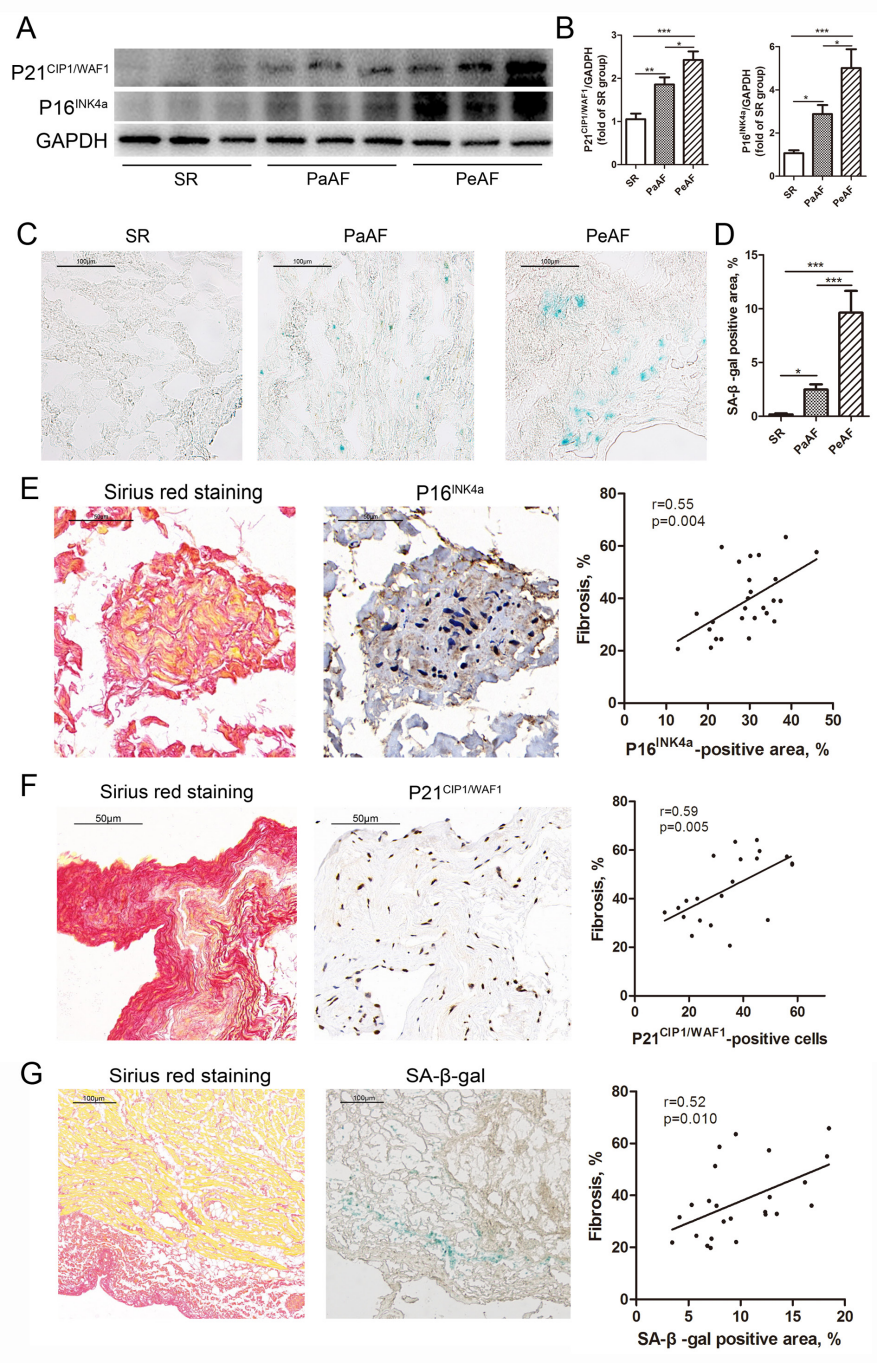
Accumulation of senescence accompanied by fibrosis in AF **A**., The expression of P21 and P16 was significantly increased in PeAF group, followed by PaAF and SR groups. **B**., Statistical analyses of P21 and P16 (*n* = 3 per group). **C**., Representative images of SA-β-gal staining in SR, PaAF and PeAF groups. **D**., Quantification of SA-β-gal positive area in the three groups (*n* = 5 per group). **E**., Left, representative images of Sirius red staining and immunohistochemical staining for P16; bar = 50μm. Right, linear analysis of P16- and Sirius red-positive area (*n* = 26). **F**., Left, representative images of Sirius red staining and immunohistochemical staining for P21; bar = 50μm. Right, linear analysis of P21-postive cells and Sirius red-positive area (*n* = 21). **G**., Left, representative images of Sirius red staining and SA-β-gal staining; bar = 100μm. Right, linear analysis of SA-β-gal- and Sirius red-positive area (*n* = 24). Values were expressed as mean ± SD. *P < 0.05, **P < 0.01, ***P < 0.001.

**Figure 3:**
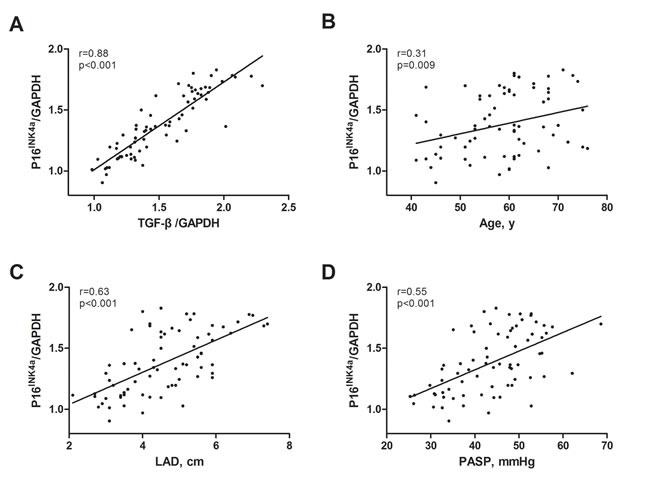
Correlation of P16 with clinical parameters **A**., P16 was positively associated with TGF-β, *n* = 72. **B**., P16 was positively associated with age, *n* = 72. **C**., P16 was positively associated with left atrial diameter (LAD), *n* = 72. **D**., P16 was positively associated with pulmonary artery systolic pressure (PASP), *n* = 72.

A positive correlation was seen between the expression of senescence markers and fibrosis in the human heart [10]. Therefore, we evaluated the expression of senescence markers, SA-β-gal, P21^CIP1/WAF1^ and P16^INK4a^ in left atrial appendages (LAAs), and tried to explore the association of senescence and fibrosis. Figure 2A and 2B presented western blotting analysis results of P21^CIP1/WAF1^ and P16^INK4a^. Results revealed significant increase of P21^CIP1/WAF1^ and P16^INK4a^ in the PeAF group, followed by PaAF and SR groups. SA-β-gal staining results also demonstrated highest expression of SA-β-gal in PeAF group (Figure 2C and 2D). We additionally performed linear correlation analysis to determine the relationship between senescence and fibrosis. According to the results of western blotting, P16^INK4a^ expression was positively associated with TGF-β expression (*r* = 0.88, *p* < 0.001, Figure 3A). Similarly, positive area of senescence markers, SA-β-gal and P16^INK4a^, was correlated positively with fibrotic lesions (Figure 2E-2G).

### Correlation between P16^INK4a^ expression and clinical parameters

Senescent cells accumulate with aging, and senescence is considered as an “antagonistic” hallmark of aging [7]. So, we evaluated the association of P16^INK4a^ expression with age. As illustrated in Figure 3B, P16^INK4a^ expression was positively associated with age (*r* = 0.31, *p* = 0.009). Since LAD and PASP were higher in the AF groups and similarly the expression of P16^INK4a^, we also investigated the relationship between them. Figure 3C and 3D showed that P16^INK4a^ level was positively and significantly correlated with LAD and PASP (*r* = 0.63, *p* < 0.001 and *r* = 0.55, *p* < 0.001, respectively).

### CFs and premature senescence

**Figure 4:**
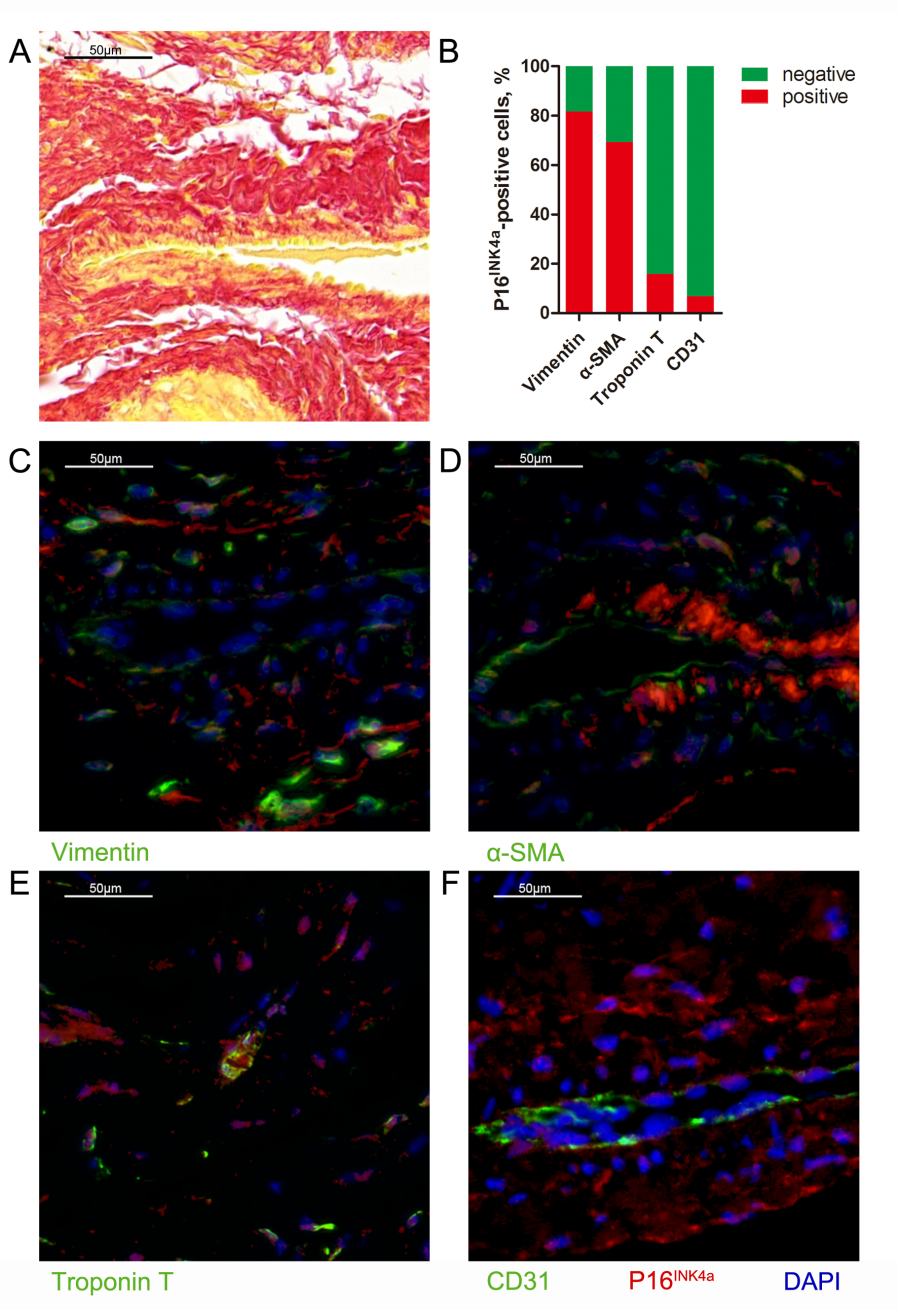
Cardiac fibroblasts are the predominant cells experiencing premature senescence in human heart **A**., Representative image of Sirius red staining for perivascular fibrosis (bar = 50μm). **B**., Quantification of P16-positive cells in perivascular area that express vimentin, α-smooth muscle actin (SMA), troponin T and CD31. **C**., Representative image of co-stained for P16 (red) and vimentin (green) (bar = 50μm). **D**., Representative image of co-stained for P16 (red) and α-SMA (green) (bar = 50μm). **E**., Representative image of co-stained for P16 (red) and troponin T (green) (bar = 50μm). **F**., Representative image of co-stained for P16 (red) and CD31 (green) (bar = 50μm).

Our results showed that accumulation of senescent cells were accompanied with fibrotic lesions, suggesting that CFs may be predominant in the senescent cells. Therefore, we conducted immunofluorescent assay to identify the type of senescent cells in the fibrotic lesions. As shown in Figure 4B-4D, majority of the senescent cells (P16^INK4a^-positive) expressed the fibroblast marker, vimentin (81.6±3.7%). While 69.3±8.5% of P16^INK4a^-positive cells expressed the myofibroblast marker α-smooth muscle actin. However, only 15.8±2.1% and 6.7±2.8% of senescent cells expressed troponin T (cardiomyocyte marker) and CD31 (endothelial cell marker). These data suggested that premature senescence of CFs and transformation of CF to myofibroblasts are associated with AF development.

### P16^INK4a^/TGF-β in patients with AF recurrence

It is reported that fibroblast senescence plays an essential role as antifibrotic in myocardial fibrosis [10, 11]. Thus, we hypothesized that senescence might have predictive value in the recurrence of AF. All patients with PeAF underwent successful surgical ablation. During a 12-month follow-up period, 20 patients (65.6%) restored SR, while other 10 (34.4%) patients had recurrence of AF (Table 2). Patients with recurrence of AF demonstrated significant increase in LAD compared with SR patients (6.1±1.1 *VS* 5.1±0.8 cm, *p* = 0.018). No statistical difference was observed for other clinical parameters among the groups. Similar to LAD, the expressions of P16^INK4a^ and TGF-β were higher in the recurrence group than in the non-recurrence group (1.73±0.05 *VS* 1.61±0.11, *p* = 0.004 and 2.01±0.18 *VS* 1.73±0.13, *p* < 0.001, respectively). Conversely, the relative expression of P16^INK4a^/TGF-β was decreased in patients with AF recurrence (0.87±0.07 *VS* 0.93±0.07, *P* = 0.026), indicating that premature senescence may have an antifibrotic effect on the development of AF.

**Table 2:** Characteristics of PeAF followed up for one year

Parameters	Non-recurrence (*n*= 20)	Recurrence (*n*= 9)	*P* value
Basic data			
Gender (M/F), *n*	7/13	4/5	0.628
Age, y	59.1±7.8	61.8±10.0	0.441
BMI, kg/m^2^	24.5±3.1	26.1±3.7	0.252
NYHA class	2.7±0.6	2.2±1.0	0.101
ECG parameters			
LVEDd, cm	5.1±0.8	5.5±1.1	0.331
LVESd, cm	4.2±0.8	4.6±1.0	0.255
LAD, cm	5.1±0.8	6.1±1.1	0.018
LVEF, %	56.6±7.9	52.1±7.3	0.165
PASP, mmHg	48.2±6.4	51.9±7.8	0.189
Preoperative drugs			
ACEI/ARB, *n*	4	0	0.148^#^
β-blockers, *n*	4	2	0.891^#^
CCB, *n*	4	0	0.148^#^
Digoxin, *n*	17	7	0.634^#^
Spirolactone, *n*	8	5	0.436^#^
Protein expression			
P16^INK4a^/GAPDH	1.61±0.11	1.73±0.05	0.004
TGF-β/GAPDH	1.73±0.13	2.01±0.18	<0.001
P16^INK4a^/TGF-β	0.93±0.07	0.87±0.07	0.026

Abbreviations as in Table 1.

#Fisher's exact test.

## DISCUSSION

In the present study, we evaluated the expression of senescence markers, SA-β-gal, P21^CIP1/WAF1^ and P16^INK4^, in LAAs from patients with AF. We demonstrated that premature senescence of CFs was increased and accumulated, and was accompanied by fibrosis in AF patients. Moreover, lower ratio of P16^INK4a^/TGF-β predicted the recurrence of AF after ablation. Taken together, these data established senescent CFs as potential mediators for cardiac fibrogenesis and AF development, and revealed premature senescence as a potential antifibrotic factor in AF.

AF is strongly age dependent, and it affects approximately 1%, 4% and 15% at 50, 65 and 80 years, respectively [3]. Mounting evidence suggest that extracellular matrix (ECM) and perivascular fibrosis were increased progressively with age, leading to cardiac remodeling and dysfunction in elderly individuals [12]. Besides, telomere attrition affects mitochondrial function, thus promoting aging [13], and short telomere length is considered to be a hallmark of aging [14]. Recently, Carlquist et al found that AF subjects had shorter telomeres compared with SR subjects [6]. These evidence suggest that aging, also called replicative senescence, contributes to the development and maintenance of AF. In contrast, premature senescence involves growth-arrest that limits the proliferation of mammalian cells and eliminates the damaged cells [7]. Senescent cells are characterized by up-regulation of P16^INK4a^, P14^ARF^ (P19^ARF^ in mice), P21^CIP1/WAF1^, P53, and SA-β-gal activity [7, 10, 11, 15, 16]. Also, senescent cells exhibit the up-regulation of senescence-associated secretory phenotypes (SASP), such as IL-6, IL-8, MCP-1 and TNF-α, and generates an inflammatory microenvironment that may lead to the clearance of senescent cells [7, 16]. In the present study, we found increased expression of senescence markers, P21^CIP1/WAF1^ and P16^INK4^ and SA-β-gal in AF patients with valvular heart diseases. We also identified CFs as predominant cells that experience senescence. Our results supported the hypothesis that premature senescence of CFs is increased during the progression of AF.

Moreover, the results of Sirius red staining found an increase in atrial fibrosis in both the PaAF and PeAF groups. Atrial fibrosis exhibited excessive deposition of ECM, comprising of Col I and Col III [12]. The ratio of Col I/ Col III represents myocardial stiffness that can induce atrial fibrosis [17]. Additionally, TGF-β is established as a profibrotic factor that plays a key role in atrial remodeling of AF [18]. Therefore, we detected the expression of Col I, Col III and TGF-β, and also calculated the ratio of Col I and Col III. In accordance with the previous studies [17, 19], up-regulation of Col I, Col III, TGF-β and increased Col I/ Col III ratio were found in AF groups, especially in the PeAF group. Further analyses showed that P16^INK4a^ and SA-β-gal were positively correlated with atrial fibrosis in LAAs from patients with valvular diseases. These data suggest that premature senescence was associated with AF development with advanced atrial fibrosis.

In the year 2013, Zhu et al reported that P53-mediated fibroblast senescence increases collagen deposits after myocardial ischemia, and inhibition of P53 enhances collagen deposition and cardiac fibrosis [11]. Based on the findings of pro-senescent treatments that can reverse the skin and liver fibrosis [20, 21], there might be a possibility that premature senescence can also restrain cardiac fibrosis. Recently, Meyer et al showed that senescent fibroblasts accumulate in myocardial fibrotic tissue in mice undergoing transverse aortic constriction, and CCN1-triggered senescence of CFs limits fibrosis and in turn improves heart function in mice. Collectively, premature senescence of CFs might be a potential therapeutic target to control fibrosis. Therefore, we speculated that premature senescence might predict the recurrence of AF after surgery ablation. P16^INK4a^ and TGF-β expressions were both higher in the recurrence group than in the non-recurrence group (Table 2). The ratio of P16^INK4a^/TGF-β was lower in the recurrence group. These findings indicate that premature senescence may have an antifibrotic effect in human heart and may in turn reduce the risk of developing AF. However, further studies are warranted to confirm this conclusion.

Cellular senescence is dependent on P53/P21 and P16/P14 (P19 in mice) pathways. However, the upstream signaling factors and mechanisms of premature senescence that premature senescence of CFs restrains fibrosis in heart remain unclear. The matricellular protein, CCN1 can be used to induce fibroblast senescence both *in vitro* and *in vivo* [10, 20], and binds to integrin α6β1 and heparan sulfate proteoglycans, activating the RAC1-NOX1 complex to induce accumulation of ROS. Consequently, ROS activates P53 and triggers P16 *via* ERK/MAPK pathway, leading to cellular senescence. In addition, several mechanisms may explain the cardioprotective effect of CFs senescence. Firstly, senescence reduces the number of ECM-producing cells due to cell-cycle arrest of CFs. Secondly, production of SASP by senescent cells enhances chemokine-mediated macrophage recruitment, leading to the clearance of senescent CFs [16, 22]. Thirdly, ECM-related genes are down-regulated, while ECM-degrading enzymes are up-regulated in the senescent cells [7, 10]. However, the underlying mechanisms await further investigation.

Several limitations must be acknowledged in this study. Firstly, senescent cells accumulate with aging and can be found in aged tissues, and advanced aging might impair the senescence-induced elimination of damaged cells [7]. In the present study, we also found a positive association between age and the expression of P16^INK4a^. However, we could not clearly evaluate their interaction in this context. Secondly, all experiments were conducted in human heart specimens. We could not use genetic or pharmacological methods to investigate a causal relationship between premature senescence of CFs and AF progression. We only found that patients with AF recurrence after ablation showed lower ratio of P16^INK4a^/TGF-β, which might imply a potential antifibrotic role of senescence in AF. Thirdly, all patients enrolled in this study were with valvular heart diseases and underwent valve replacement surgery. Our results may not be generalized to the whole AF population. And the sample size of the present studies is relatively small, further studies with more patients are warranted to test our hypotheses.

In conclusion, premature senescence of CFs is associated with atrial fibrosis in AF, and may have an antifibrotic role during the occurrence of AF in patients with valvular diseases. The underlying molecular mechanisms involved in the restriction of atrial fibrosis by senescent CFs still needs confirmation.

## MATERIALS AND METHODS

### Patients and tissue specimens

A total of 72 consecutive patients experiencing valvular heart diseases undergoing valve replacement surgery were enrolled at Huai’an First People's Hospital from Jan 2015 to Dec 2016. Patients with thyroid dysfunction, chronic obstructive pulmonary disease, renal disorders, and detected rheumatic activity were excluded from the study. Besides, patients aged > 80 years or < 18 years old were also excluded. The valve replacement operations and the modified Cox maze III procedures were performed as described previously [19]. This study was approved by the ethics committee of Huai’an First People's Hospital. All patients involved signed the informed consent.

All the 72 patients were divided into three groups: SR group (*n* = 26), PaAF (*n* = 17, AF lasting < 7 days), and PeAF (*n* = 29, AF lasting > 7 days). Each patient had routine transthoracic echocardiographic examination. All patients undergoing surgical ablation were followed up for 12 months. The diagnostic recurrence of AF was based on 24-hour ambulatory electrocardiogram monitoring.

The LAA tissues were obtained prior to the establishment of extracorporeal circulation. One part of the LAA was fixed in 4% formalin for immunohistochemistry and Sirius red staining, and the remaining part was frozen in liquid nitrogen and stored at -80°C for other analyses.

### Western blotting

As described previously [23], equal amounts of protein samples were separated by sodium dodecyl sulfate polyacrylamide gel electrophoresis and transferred onto polyvinylidene difluoride membranes. The membranes were incubated with primary antibodies against GAPDH (Bioworld Technology), P21^CIP1/WAF1^, P16^INK4^ (Abcam), Col I, Col III, and TGF-β (ABclonal) at 4°C for overnight, and then were incubated with secondary antibodies at room temperature for 1 hour. Proteins were visualized using enhanced chemiluminescence kit (Millipore Corporation) and quantified by Image Pro Plus software.

### Immunohistological analyses

Paraffin-embedded sections were washed with PBS for 3 times and blocked with 1% bovine serum albumin for 30 min, and then were incubated with anti-P16 ^INK4a^ or anti-P21^CIP1/WAF1^ for overnight at 4°C. After washing for 3 times with PBS, the sections were incubated with secondary antibodies for 30 min and then counterstained with hematoxylin. To distinguish the cellular type that is experiencing premature senescence, we performed double immunofluorescence staining of senescence marker P16^INK4a^, together with vimentin, α-smooth muscle actin, troponin T (SANTA) and CD31 (Abcam) to characterize CFs, myofibroblasts, cardiomyocytes and endothelial cells, respectively.

### Sirius red staining

After fixation with paraffin, heart samples were subjected to alcoholic dehydration. The 4 μm sections were then incubated with 0.1% Sirius red solution for 45 minutes. Representative images were analyzed by Image Pro Plus software.

### SA-β-gal activity assay

The LAA tissues were stained to determine SA-β-gal activity using Senescence Detection Kit (Abcam) according to the manufacturer's instructions. Briefly, OCT-embedded LAA sections were fixed in 2% formaldehyde containing 0.2% glutaraldehyde for 15 minutes, and then were incubated with 1mg/ml X-gal for overnight at 37°C. The SA-β-gal positive area appeared green in color and was analyzed by Image Pro Plus software.

### Statistical analysis

Data were presented as mean±standard deviation for continuous variables and percentages for categorical variables. *Logarithmic transformation of* the echocardiography data *was applied to fulfill the requirement of normal distribution based on the results of* Kolmogorov-Smirnov test [24]. Differences among the groups were analyzed using analysis of variance or chi-square analysis. Generalized linear model was employed to determine the association of senescence with other parameters. SPSS version 22.0 (IBM SPSS, Armonk, NY) was used for statistical analyses. Statistical significance was considered when a 2-tailed *p* value was < 0.05.

## Abbreviations

AF: Atrial fibrillation
SA-β-gal: senescence-associated β-galactosidase
CF: cardiac fibroblast
LAD: left atrial diameter
SR: sinus rhythm
PaAF: paroxysmal atrial fibrillation
PeAF: persistent atrial fibrillation
PASP: pulmonary artery systolic pressure
ECM: extracellular matrix
SASP: senescence-associated secretory phenotype
LAA: left atrial appendage
